# Short- and long-term stability of synthetic cathinones and dihydro-metabolites in human urine samples

**DOI:** 10.1007/s11419-024-00684-2

**Published:** 2024-03-30

**Authors:** Abdulaziz A. Aldubayyan, Erika Castrignanò, Simon Elliott, Vincenzo Abbate

**Affiliations:** 1 Department of Analytical, Environmental & Forensic Sciences, Faculty of Life Sciences & Medicine, King’s College London, London, UK; 2 Department of Toxicology, Central Military Laboratory and Blood Bank, Prince Sultan Military Medical City, Riyadh, Saudi Arabia; 3 Elliott Forensic Consulting, Birmingham, UK

**Keywords:** Synthetic cathinone, Stability, Urine, NPS, LC–MS/MS

## Abstract

**Purpose:**

Synthetic cathinones constitute the second largest group of new psychoactive substances, which are often used for recreational purposes and reported in toxicological analysis. Various factors may influence the stability of synthetic cathinones between sampling and analysis, and therefore, stability studies are required to determine the best storage conditions as well as extend the period of detection.

**Methods:**

This study involved sixteen synthetic cathinones and ten dihydro-metabolites spiked in human urine to evaluate the stability under common storage conditions to imitate real forensic toxicology samples. The samples were stored at either room temperature (22–23 °C) for up to 3 days, refrigerated (4 °C) for up to 14 days or frozen (–40 °C) for up to 12 months, and analyzed in triplicate using a validated liquid chromatography–tandem mass spectrometry method.

**Results:**

Analytes’ concentrations decreased over time, although slower when stored frozen. All analytes remained stable (> 80%) for 1 month when stored frozen before losses in content were more apparent for some compounds, depending on their chemical structure. Under all storage conditions, the highest instability was observed for analytes containing halogens (i.e., chlorine or fluorine). Thus, halogenated analytes were further investigated by using liquid chromatography coupled to quadruple time-of-flight mass spectrometry to attempt identifying degradation products.

**Conclusions:**

Irrespective of parent analytes, dihydro-metabolites had improved stability at each tested temperature, which highlights their importance as appropriate urine biomarkers when retesting is required after a long period of storage.

**Supplementary Information:**

The online version contains supplementary material available at 10.1007/s11419-024-00684-2.

## Introduction

Synthetic cathinones (SCt) are one of the largest groups of new psychoactive substances (NPS) in illicit drug markets [1]. Their inexpensive cost and ease of availability via online vendors are assumed to contribute to their expansion and popularity among drug users [2].

An extensive period of time before analysis may be needed when laboratories are encountered with backlogs or when samples must be retained over a long period of time to allow retesting (if requested). Hence, knowledge of their stability is of great importance to assist the interpretation of the results as some analytes may degrade during sample storage. Stability may be influenced by the storage conditions, chemical structure, and/or type of biological samples. These factors must be taken into account when comparing initial results and results after days, weeks, and months of storage.

A number of studies indicate instability of some SCt in biological samples, with the storage temperature being an important factor [3]. Al-Saffar et al. [4] studied the stability of methcathinone, buphedrone, mephedrone, 3-fluoromethcathinone (3-FMC), 4-fluoromethcathinone (4-FMC), methedrone, methylone, butylone, pentylone, methylenedioxypyrovalerone (MDPV), and naphyrone in urine samples maintained at − 20 °C (freezer), 6 °C (refrigerator), and 22 °C (room temperature (RT)) for 3 months. Degradation of analytes was higher at 22 °C than at 6 °C but less pronounced at –20 °C.

In addition to temperature, mechanisms of degradation can also be influenced by chemical structure and urinary pH. Glicksberg and Kerrigan [5] investigated the stability of 22 SCt in acidic (pH 4) and alkaline (pH 8) urine samples at –20 °C, 4 °C, 20 °C, and 32 °C for a period of 6 months. They found improved stability of tertiary amines SCt incorporating methylenedioxy (MD) groups over other tertiary amines-containing SCt, followed by secondary amines. Among secondary amines, flephedrone and its isomer 3-fluoromethcathinone (3-FMC) exhibited the worst stability at pH 8 even when maintained at –20 °C and preserved (1% sodium fluoride), while stability improved in those analytes at pH 4. Adamowicz and Malczyk [6] corroborated these findings when they reported that instability of SCt increase with the increasing pH.

Parent SCt share a carbonyl functional group in their chemical structure. This carbonyl is readily reduced to a hydroxyl group, leading to the so-called dihydro-metabolites [7]. It has been recommended that stability studies should involve both parent and major metabolites, as this may be helpful to establish a clear image of changes in drug concentrations and degradation [8]. While urine samples are typically characterized by the presence of metabolites, which can aid in determining the intake of the parent drug, most published reports on the stability of SCt mainly focused on the stability of parent drug [3]. A literature search only identified two published studies on the stability of SCt metabolites in urine. In a study performed by Concheiro et al. [9] dihydro-4-methylethcathinone (4-MEC), dihydro-buphedrone, and dihydro-mephedrone were found to be stable at RT for 24 h and at 4 °C for 72 h. In contrast, other authors have reported dihydro-4-MEC as being unstable in urine samples after 24 h of storage at 4 °C, whereby degradation of 22% from the initial SCt level was found [10]. With respect to other biological matrices, dihydro-mephedrone and dihydro-nor-mephedrone (one of the main phase I mephedrone metabolites) were consistently stable in whole blood for 10 days at either 4 and –20 °C, whereas a decrease up to 23.4% was observed in the corresponding parent cathinone at 4 °C [11]. Soh and Elliott [12] investigated the stability of 4-MEC in blood and plasma and found that dihydro-4-MEC was the prominent metabolite in the absence of 4-MEC (for instance via in vitro and ex vivo sample instability). In our previous work, dihydro-metabolites were more stable than their respective parent drugs in whole blood over 6 months regardless of storage temperatures [13]. Similar stabilities might be expected in urine samples, but this information appears to be scarce in the literature. Therefore, it is important to assess the stability of metabolites as they may be considered more appropriate biomarkers if the parent drugs are extensively degraded.

**Fig. 1:**
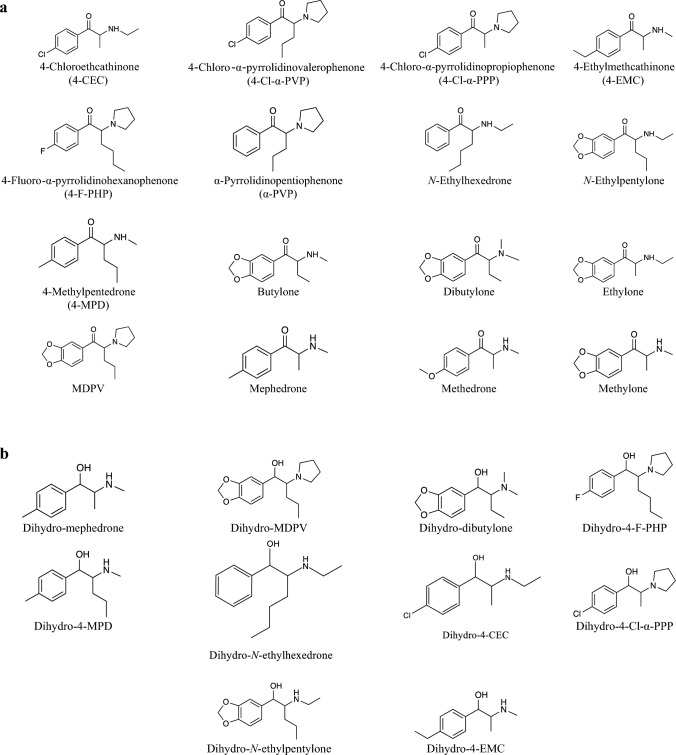
Chemical structures of synthetic cathinones **a** and dihydro-metabolites **b** involved in this study

The purpose of this study was to investigate the stability of a panel of SCt and dihydro-metabolites in human urine samples under the typical conditions used in forensic toxicology. Storage conditions included RT for 3 days, 4 °C for 14 days, and –40 °C over 12 month. A recently reported fully validated liquid chromatography–tandem mass spectrometry (LC–MS/MS) method was used to assess the stability of a total of 26 analytes (Fig. 1) at each storage condition [14].

## Materials and methods

### Chemicals and reagents

Methanol and acetonitrile (ACN) were obtained from Sigma-Aldrich (Dorset, UK). All solvents used were high-performance liquid chromatography (HPLC) grade or better. The following standards were purchased as hydrochloride salts from Chiron (Surrey, UK): 4-CEC, 4-EMC, butylone, dibutylone, ethylone, mephedrone, methedrone, MDPV, methylone, *N*-ethylhexedrone, *N*-ethylpentylone and α-PVP. Corresponding internal standard MDPV-*d*_8_ (hydrochloride) was obtained from Sigma-Aldrich (Dorset, UK). Tic TaC communications (London, UK) kindly donated 4-Cl-α-PPP, 4-F-PHP, 4-MPD, and 4-Cl-α-PVP. Ultra-pure water (18.2 MΩ) was generated using a Millipore water system (Burlington, MA, USA). Two milliliters Eppendorf capped tubes were obtained from Eppendorf (Stevenage, UK).

As described in [14], dihydro-metabolites were synthesized in-house, and the identity and purity of the synthesized materials were assessed via electrospray ionization (ESI)-MS and ESI-MS/MS (direct infusion). Initial analyses were performed for dihydro-mephedrone, the metabolite structural information of which has been previously described in detail [11, 15], before the identity and purity of all other synthesized metabolites were examined. Primary analysis of reduced mephedrone by ESI-MS via direct infusion resulted in a major signal at *m/z* 180 [M + H]^+^. The identity of the reduced metabolite was further confirmed by scanning for product ions of the *m/z* 180 [M + H]^+^ precursor ion using ESI-MS/MS (direct infusion). The mass spectrum of the product scan shows the most intense product ions of *m*/*z* 162 and 147. Another minor product ion observed was *m*/*z* 131, which corroborate what had been seen for dihydro-mephedrone in previous studies [11, 15]. To ensure complete reduction of mephedrone to dihydro-metabolite, the presence of mephedrone precursor ion (*m/z* 178 [M + H]^+^) and characteristic fragments in the spectrum was further assessed via direct infusion/ESI-MS and ESI-MS/MS analysis. The synthesized metabolite contained no parent drug (i.e., mephedrone) shown by the absence of a signal at *m/z* 178 [M + H]^+^, and no product ions with *m*/*z* of 160 and 145 were detectable either. Thus, it seemed reasonable to assume that the synthesized metabolite was pure indicated by the absence of the parent peaks. To further confirm the identity, the synthesized material was subjected to full scan high-resolution mass spectrometry in addition to MS/MS scan, which resulted in the protonated molecular ion and product ions matching to those observed for dihydro-mephedrone [11, 15].

### Stability study design

Human drug-free urine samples were obtained from 10 different donors based on a protocol approved by the Research Ethics Committee at King’s College London (HR-18/19- 10507). All unpreserved urine samples were pooled to produce a single urine sample. Urinary pH was measured using a pH meter (pH 7.63). Pooled urine samples were then fortified with drug standard solutions to a final concentration of 800 ng/mL and 0.5 mL aliquots were transferred to 2 mL Eppendorf capped tubes. The 800 ng/mL fortified concentration was chosen since it falls within average concentrations encountered in urine from toxicological casework [16]. After mixing, three aliquots were immediately extracted and analyzed to establish day zero concentration (T_0_). The extraction procedure was the same as previously reported [14].

Short-term stability was evaluated after 3 and 14 days at RT and 4 °C, respectively, to replicate different situations of sample handling and transportation. Long-term stability of analytes was evaluated at –40 °C for a period of 12 month over different sampling points (7, 14, 30, 90, 185, 210, and 365 days); however, data for day 210 were unavailable due to instrument issues. To perform the stability study, three aliquots from each storage temperatures were extracted and analyzed in each sampling point.

The average concentrations of T_0_ in nanograms per milliliter were normalized to 100% and used as a baseline for the stability experiments. Thereafter, the subsequent sampling points were compared with the baseline values (determined at the establishment of the experiment), and the percentage remaining for each SCt was determined. Percentage differences from the T_0_ and the measured sampling point of each analyte were regarded unstable when values decreased > 20% of the T_0_.

### LC–MS/MS analysis

Sample analysis was performed on an Acquity UPLC^®^ system (Manchester, UK) coupled to a Waters Quattro Premier XE™ Triple Quadrupole (QqQ) Mass Spectrometer System (Manchester, UK) utilizing ESI. Data acquisition and analysis were performed using MassLynx v. 4.1 (Waters). The ion source was operated in positive mode. Two selective transition ions in multiple reaction monitoring (MRM) were used for parent analytes and one MRM for metabolites. Chromatographic separation was achieved with a HSS T3 UPLC™ analytical column (150 × 2.1 mm, 1.8  μm) (Waters) kept at a temperature of 20 °C and the pump was operated at a flow rate of 0.3 mL/min. Mobile phase A consisted of 0.1% formic acid in water and mobile phase B consisted of 0.1% formic acid in ACN. The gradient program was as follows: 0–1.80 min, 90% A; 1.80–6.0 min, 64% A; 6.0–9.80 min, 64–0% A; 9.80–10.80 min, 0% A; 10.80–10.81 min, 0–90% A; and 10.81–13.00 min, 90% A. An injection volume of 10  μL was used. The total run time was 13 min. Other LC–MS/MS parameters such as retention time, precursor ion, and product ion were described in the previously published literature [14].

### LC–QTOF-MS analysis

To investigate potential degradation products/metabolites via instability, selected analytes were spiked at 800 ng/mL in urine and then stored at RT for 3 days. Analysis was performed using an Infinity 1290 ultra-high-performance liquid chromatography system (Agilent Technologies, Santa Clara, USA), equipped with a binary solvent system, a thermostat autosampler and a thermostat column compartment. Separations were carried out on Eclipse Plus C18 column (50 × 2.1 mm, 1.8  μm) (Agilent Technologies), which was maintained at 30 °C during the analysis. Mobile phases A and B consisted of 0.1% formic acid in water and 0.1% formic acid in ACN, respectively, which were held at a flow rate of 0.3 mL/min using the following gradient elution. The gradient started at 90% A, maintained for 1.80 min, then increased to 64% B until 6.0 min, and further increased to 100% B until 8.00 min. The gradient finally returned to 90% A at 8.01 min until 9.00 min to re-equilibrate the column. An injection volume of 1.0 μL was used.

Mass spectrometry analysis was performed using Agilent 6545XT AdvanceBio quadruple time-of-flight (QTOF) system (Agilent Technologies), equipped with a dual Agilent jet stream ESI source (dual AJS ESI). The mass spectrometer was operated in positive ionization mode with gas temperature set at 150 °C; drying gas 12 L/min; nebulizer pressure 25 psi; sheath gas temperature and flow that were 350 °C and 12 L/min, respectively; fragmentor voltage 140 V; capillary voltage 3500 V, and skimmer voltage 65 V. Internal reference masses of 121.050873 (purine) and 922.009798 (HP-0921) were used. The mass spectrometer was set to auto MS/MS mode. Collision energies of 10–40 eV were used for fragmentation. MassHunter^®^ Qualitative Analysis software (version B.08.01, Agilent Technologies) was used for data processing.

## Results

**Fig. 2:**
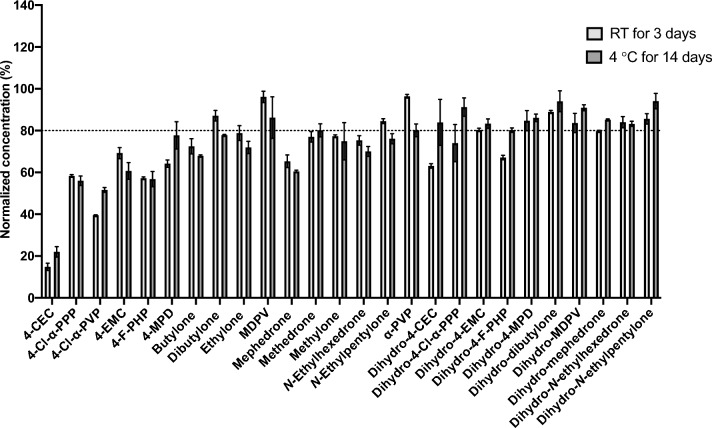
Short-term stability study of SCt analytes and metabolites in urine samples following storage at RT for 3 days and at 4 °C for 14 days. Data are presented as % remaining compared to T_0_. Error bars represent standard deviation among triplicate measurements (*n* = 3)

**Fig. 3:**
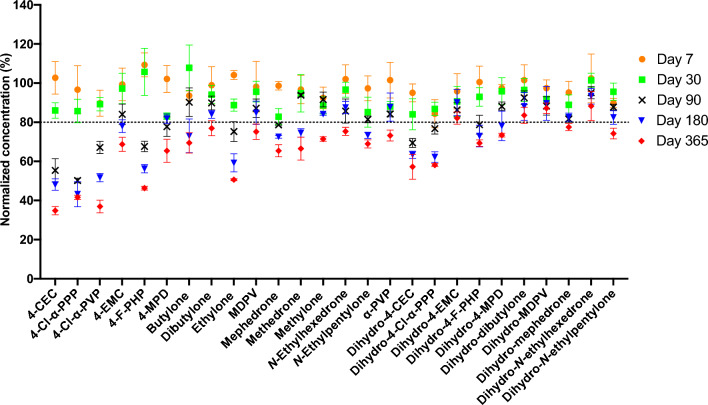
Long-term stability study of SCt analytes and metabolites in urine samples following storage at –40 °C for 12 months. Data are presented as % remaining compared to T_0_. Error bars represent standard deviation among triplicate measurements (*n* = 3)

The stability data expressed as the percentage remaining of each tested analyte after 3 days at RT and 14 days in refrigerated (4 °C) (Fig. 2) and over 12 month in frozen (–40 °C) storage conditions are summarized in Fig. 3.

For short-term stability under both RT and at 4 °C, most of the SCt displayed stability issues. 4-CEC had less than 15% of its initial concentration remaining after three days when kept at RT, and only 22% remaining when kept in the refrigerator. While the percentage concentrations remaining of 4-Cl-α-PPP, 4-Cl-α-PVP and 4-F-PHP were more than 40% when stored at RT, refrigerating the samples resulted in more than 50% intact analytes for 14 days. The rest of the SCt that were kept at RT or 4 °C exhibited stability differences within the study period, but the overall remaining concentration was more than 60%. Among all the tested SCt, MDPV (*N*-pyrrolidine SCt with a MD group) displayed the greatest stability in which more than 80% of the initial concentration remained after 3 and 14 days when stored at RT and 4 °C, respectively. α-PVP showed similar behavior to MDPV.

Contrary to parent drugs, most dihydro-metabolites, except for dihydro-4-CEC, dihydro-4-Cl-α-PPP, and dihydro-4-F-PHP (which had more than 60% remaining), were stable after 3 days and 14 days at RT and 4 °C, respectively.

Concerning long-term stability (Fig. 3), freezing the urine samples maintained good stability across all parent drugs for up to 1 month of storage. On day 90, parent analytes demonstrated minimal variation within the stability criteria where the overall loss was less than 25% of the original contents, except for halogenated analytes that experienced significant degradation, especially 4-CEC and 4-Cl-α-PPP, in which only about 50% of their initial values remained detectable. With the exception of halogenated analytes (recoveries of about 40–55%), the concentration remaining for all parent drugs did not decrease by more than 65% after 6 months of storage and was relatively maintained to these levels until the end of the study period.

The greatest stability was noticed with all dihydro-metabolites when stored frozen. Even though instability was observed to some extent in some metabolites (e.g., dihydro-4-MPD, dihydro-mephedrone, dihydro-*N*-ethylpentylone), particularly after 6 months and onwards, the maximum loss did not exceed 30% of the total content. All other metabolites were found to be fairly stable under this condition, as more than 80% of their content was still detectable after 12 months of storage, with the exception of halogenated SCt: dihydro-metabolites of 4-CEC, 4-Cl-α-PPP, and 4-F-PHP were found to be unstable with about 70% remaining after 90 days of storage, reaching the maximum of about 60% remaining after 12 months of storage. Nevertheless, in comparison to their respective parent drugs, dihydro-metabolites were overall more stable.

**Fig. 4:**
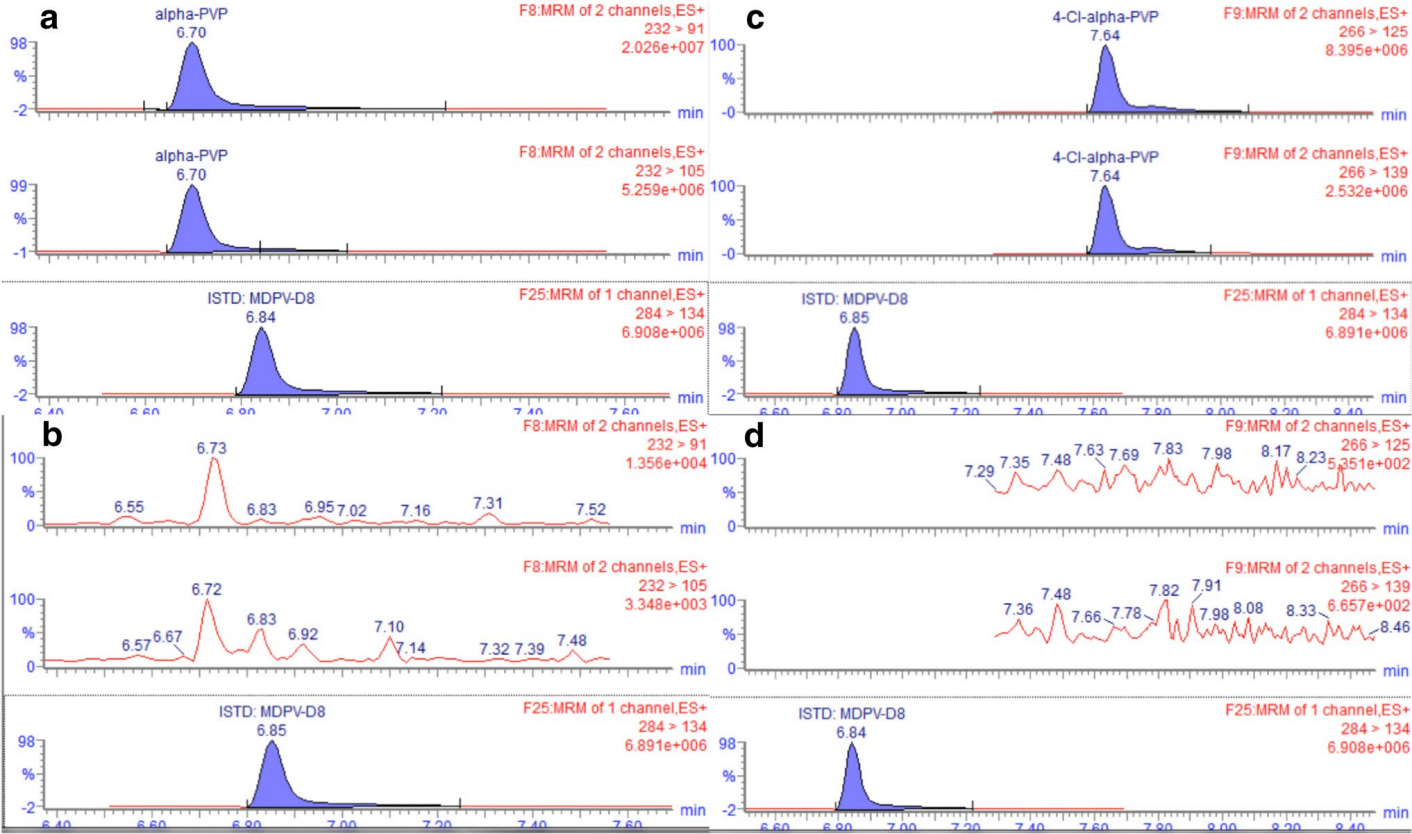
MRM windows of α-PVP **a**; 4-Cl-α-PVP **b**; α-PVP following separate run of 4-Cl-α-PVP in urine extract **c**; and MRM windows of 4-Cl-α-PVP following separate run of α-PVP **d**

Given both α-PVP and 4-Cl-α-PVP were part of the tested panel, each analyte was fortified separately and stored at RT for 3 days before separately run following the procedure described in “stability study design” section. Visual inspection of the MRM chromatogram of α-PVP following analysis of 4-Cl-α-PVP revealed a peak at the same RT (6.72 min) for the MRM transitions m/z 232 > 91 and m/z 232 > 105 (Fig. 4c). Of note, the reverse was not observed (Fig. 4d).

## Discussion

Information on extended stability is of paramount importance since it may address the maximum storage time of a drug in a particular biological matrix. In addition to parent analytes, this study incorporated a selection of dihydro-metabolites that have been recently detected in toxicological casework – either with or without their respective parent drug [14, 17] – but where stability considerations are lacking in the literature. This study presents, for the first time, the stability data of SCt and dihydro-metabolites over a much longer period of time in human urine samples.

The influence of storage temperature appears to be more significant for halogenated analytes, which are typically the most unstable SCt in different matrices [3]. This observation has been previously reported and has been attributed to the presence of a halogen atom (e.g., chlorine or fluorine) on the benzene ring that adversely affects SCt stability [6, 13]. In parallel, mephedrone, one of the most commonly detected SCt in toxicology casework [18] had also significant loss at RT and 4 °C storage conditions, which accounted for approximately 60% of the initial mephedrone concentration. The stability of mephedrone and MDPV in urine at RT and 4 °C for up to 14 days has also been studied elsewhere [19]. Both mephedrone and MDPV were found to be fairly stable when stored refrigerated throughout the study period, but instability in mephedrone was observed at RT (about 65% remaining) following 7 days of storage [19]. While these results agree with those seen in this study for mephedrone and MDPV stored at RT, our results were contradictory with the previous work in that mephedrone concentration dropped to approximately 60% remaining after 14 days at 4 °C. These results indicate that while it is evident that the degree of degradation for most SCt stored at RT was larger than those kept at 4 °C, degradation rate was dependent on the chemical structure. Thus, these conclusions agree with those of Glicksberg and Kerrigan [5], who found that *N*-pyrrolidine derivatives are more stable than those of primary and secondary amine groups.

By demonstrating the relatively rapid degradation of certain SCt in urine samples, our analysis of dihydro-metabolites in this matrix has shown that long-term use can be monitored not only due to extensive metabolism but also because they are more stable. The different stabilities between parent and dihydro-metabolites affirm findings in Concheiro et al. [9] in which degradation of dihydro-metabolites in urine was substantially less than that of parent drugs. These results are also in line with Al-Saffar et al. [4] for the stability of SCt. That study showed most SCt were relatively stable in urine when stored at –20 °C for up to 3 months, while a higher decrease (up to 40% remaining) in concentration was observed for analytes substituted with halogen atom (i.e., 4-fluoromethcathinone and 3-fluoromethcathinone) [4].

Generally, samples containing dihydro-metabolites had an overall smaller degree of loss as opposed to parent analytes under all conditions for similar time periods. While this is the first long-term study concerning the degradation of dihydro-metabolites in urine samples, they are consistent with our previous findings on these analytes in whole blood [13], although reflecting greater stability in whole blood than in this study when stored frozen at –40 °C for 6 months. This might be explained by pH differences of both matrices (pH 7.41 in whole blood in the previous study vs. pH 7.63 in our study), which is a typically less stable medium for these analytes. In another study in urine, no degradation was found at pH 4, but when pH increased to 8, most of SCt concentrations significantly decreased after 6 months of storage at –20 °C [5]. Cook et al. [20] found a slight increase in urine pH (+ 0.7) over 2 weeks even when stored frozen. However, stability at RT and 4 °C were much higher than those in whole blood [13], suggesting the impact of other factors (e.g., enzymatic activity). Nevertheless, as stated earlier, the goal of this work was to simulate casework samples since urine pH is not commonly adjusted. As such, this stability study is probably more representative of what may be experienced in real toxicological cases.

Another crucial aspect in stability studies is the investigation of potential degradation products of target analytes, more specifically, the halogenated analytes that exerted significant degradation effects under all storage conditions, suggesting a distinct reaction may be involved in the degradation of halogenated analytes. Following analysis of 4-Cl-α-PVP, the degradation products corresponding with the *m*/*z* 232 might be attributed to α-PVP. A tentative explanation might be due to the dehalogenation of 4-Cl-α-PVP to generate α-PVP via instability.

Unfortunately, non-halogenated reference materials for 4-CEC, 4-Cl-α-PPP, and 4-F-PHP were not available at the time of the test to provide more insight into this reaction. Nevertheless, LC–QTOF-MS in full scan and auto MS/MS modes were used to attempt to identify potential 4-CEC, 4-Cl-α-PVP, 4-Cl-α-PPP, and 4-F-PHP breakdown products along with metabolites. In addition to untargeted analysis, targeted analysis was performed based on the results of previous in vivo and in vitro experiments in human biological samples, namely, carbonyl group reduction, hydroxylation, *N*-deethylation, and demethylenation [21, 22], and a list of predicted degradation products was used as potential targets (Supplementary material; Table S1). No potentially postulated degradation products were detected in any of urine samples with the present method. While it can be assumed that breakdown products were missed due to instability, the formation of undetected breakdown products in these samples, leading to analytes degradation, cannot be ignored. This hypothesis is supported by earlier suggestions that only trace amounts of dihydro-metabolites were detected via in vitro instability in whole blood and plasma [12].

## Conclusions

This study reported herein evaluated the stability of selected SCt in human urine in a more comprehensive manner due to the inclusion of metabolites and longer timeframe than previously published work. From the obtained results, the stability of SCt was highly influenced by the storage temperature, whereby storage at high temperatures accelerated the rate of degradation. Although slower when stored frozen, degradation of these analytes increased over time. Moreover, parent analytes containing halogen such as 4-CEC, 4-Cl-α-PPP, 4-Cl-α-PVP, and 4-F-PHP were characterized by extreme instability in urine samples, but marked stability differences were observed for their respective dihydro-metabolites in all storage conditions, making them potential better biomarkers of SCt use.

While –20 °C is routinely used as a frozen condition in toxicology laboratories, it is also important to test the hypothesis that deep freezing (–40 °C or lower) could be more suitable condition for urine samples suspected to contain SCt, which might help more efficiently preserve the analytes for a longer period of time. Thus, urine samples potentially containing SCt should be stored frozen and analyzed in a timely manner to avoid any significant losses.

### Supplementary Information

Below is the link to the electronic supplementary material.

Supplementary file1 (DOCX 537 KB)
