# Supplementary material for: Short- and long-term stability of synthetic cathinones and dihydro-metabolites in human urine samples
Source: Forensic Toxicol. 2024 Mar 30;42(2):172–80. doi: 10.1007/s11419-024-00684-2 (PMC11269387; doi:10.1007/s11419-024-00684-2)
Supplement: Supplementary file 1 — Supplementary file1 (DOCX 537 KB) [file 11419_2024_684_MOESM1_ESM.docx]

# Supplementary material

# Short- and long-term stability of synthetic cathinones and dihydro-metabolites in human urine samples

Abdulaziz A. Aldubayyan ^1-2^, Erika Castrignanò^1^, Simon Elliott ^1,3^, Vincenzo Abbate* ^1^

^1^Department of Analytical, Environmental & Forensic Sciences, Faculty of Life Sciences & Medicine, King’s College London, London, UK

^2^Department of Toxicology, Central Military Laboratory and Blood Bank, Prince Sultan Military Medical City, Riyadh, Saudi Arabia

^3^Elliott Forensic Consulting, Birmingham, UK

**Corresponding author: Vincenzo Abbate; vincenzo.abbate@kcl.ac.uk**

**Table S1** A list of predicted degradation pathways of 4-CEC, 4-Cl-α-PVP, 4-Cl-α-PPP and 4-F-PHP derived from *in vivo* and *in vitro* experiments in human biological samples

| Compound | Metabolic pathway | Exact mass | Ionisation mode | Elemental composition | Structure |
| --- | --- | --- | --- | --- | --- |
| **4-Chloroethcathinone (4-CEC) (parent)** | - | 212.0837 | ESI+ | C11H15ClNO+ |  |
|  | *N*-desethylation (Nor-4-CEC) | 184.0524 | ESI+ | C9H11ClNO+ |  |
|  | *N*-desethylation-β-keto-reduction | 186.0680 | ESI+ | C9H13ClNO+ |  |
|  | Oxidative dehalogenation | 194.1176 | ESI+ | C11H16NO2+ |  |
|  | *N*-desethylation-*N*-oxygenation | 200.0473 | ESI+ | C9H11ClNO2+ |  |
|  | Hydroxylation | 208.1332 | ESI+ | C12H18NO2+ |  |
|  | β-keto reduction (dihydro-4CEC) | 214.0993 | ESI+ | C11H17ClNO+ |  |
|  | Carboxylation | 222.1125 | ESI+ | C12H16NO3+ |  |
|  | *N*-oxygenation | 228.0786 | ESI+ | C11H15ClNO2+ |  |
|  | Carboxylation | 242.0578 | ESI+ | C11H13ClNO3+ |  |
| **4-Cl-**α**-PVP (parent)** |  | 266.1306 | ESI+ | C15H21ClNO+ |  |
|  | *N*,*N*-dealkylation | 212.0837 | ESI+ | C11H15ClNO+ |  |
|  | *N*,*N*-dealkylation-β-keto-reduction | 214.0993 | ESI+ | C11H17ClNO+ |  |
|  | *N*,*N*-dealkylation-*N*-oxygenation | 228.0786 | ESI+ | C11H15ClNO2+ |  |
|  | Hydroxylation | 262.1802 | ESI+ | C16H24NO2+ |  |
|  | β-keto-reduction | 268.1463 | ESI+ | C15H23ClNO+ |  |
|  | Carboxylation | 270.0891 | ESI+ | C13H17ClNO3+ |  |
|  | Carboxylation | 276.1594 | ESI+ | C16H22NO3+ |  |
|  | Carbonylation | 280.1099 | ESI+ | C15H19ClNO2+ |  |
|  | *N*-oxygenation | 282.1255 | ESI+ | C15H21ClNO2+ |  |
|  | *N*-oxygenation-β-keto-reduction | 284.1255 | ESI+ | C15H23ClNO2+ |  |
| **4-Cl-**α**-PPP (parent)** |  | 238.0993 | ESI+ | C13H17ClNO+ |  |
|  | *N*-*N*-dealkylation | 184.0524 | ESI+ | C9H11ClNO+ |  |
|  | *N*-*N*-dealkylation-β-keto-reduction | 186.0680 | ESI+ | C9H13ClNO+ |  |
|  | *N*-*N*-dealkylation-*N*-oxygenation | 200.0473 | ESI+ | C9H11ClNO2+ |  |
|  | *N*-*N*-dealkylation-β-keto-reduction-*N*-oxygenation | 202.0629 | ESI+ | C9H13ClNO2+ |  |
|  | Oxidative dehalogeation | 220.1332 | ESI+ | C13H18NO2+ |  |
|  | Hydroxylation | 234.1489 | ESI+ | C14H20NO2+ |  |
|  | β-keto-reduction | 240.1150 | ESI+ | C13H19ClNO+ |  |
|  | *N*-*N*-dealkylation-carboxylation | 242.0578 | ESI+ | C11H13ClNO3+ |  |
|  | *N*-*N*-dealkylation-β-keto-reduction-carboxylation | 244.0735 | ESI+ | C11H15ClNO3+ |  |
|  | Carboxylation | 248.1281 | ESI+ | C14H18NO3+ |  |
|  | Carbonylation | 252.0786 | ESI+ | C13H15ClNO2+ |  |
|  | *N*-oxygenation | 254.0942 | ESI+ | C13H17ClNO2+ |  |
|  | *N*-oxygenation-β-keto-reduction | 256.1099 | ESI+ | C13H19ClNO2+ |  |
| **4-F-PHP (parent)** |  | 264.1758 | ESI+ | C16H23FNO+ |  |
|  | *N*-*N*-dealkylation | 210.1289 | ESI+ | C12H17FNO+ |  |
|  | *N*-*N*-dealkylation-β-keto-reduction | 212.1445 | ESI+ | C12H19FNO+ |  |
|  | *N*-*N*-dealkylation-*N*-oxygenation | 226.1238 | ESI+ | C12H17FNO2+ |  |
|  | Oxidative dehalogenation | 262.1802 | ESI+ | C16H24NO2+ |  |
|  | β-keto-reduction | 266.1915 | ESI+ | C16H25FNO+ |  |
|  | *N*-*N*-dealkylation-β-keto-reduction-carboxylation | 268.1343 | ESI+ | C14H19FNO3+ |  |
|  | Hydroxylation | 276.1958 | ESI+ | C17H26NO2+ |  |
|  | Carbonylation | 278.1551 | ESI+ | C16H21FNO2+ |  |
|  | *N*-oxygenation | 280.1707 | ESI+ | C16H23FNO2+ |  |
|  | *N*-oxygenation-β-keto-reduction | 282.1864 | ESI+ | C16H25FNO2+ |  |
|  | Carboxylation | 290.1751 | ESI+ | C17H26NO3+ |  |
